# Antimicrobial Susceptibility and Genetic Epidemiology of Extended-Spectrum β-Lactamase-Positive Enterobacterales Clinical Isolates in Central Poland

**DOI:** 10.3390/ijms25158371

**Published:** 2024-07-31

**Authors:** Małgorzata Brauncajs, Filip Bielec, Anna Macieja, Piotr Machnicki, Dorota Pastuszak-Lewandoska

**Affiliations:** 1 Department of Microbiology and Laboratory Medical Immunology, Medical University of Lodz, 90-151 Lodz, Poland; malgorzata.brauncajs@umed.lodz.pl (M.B.); anna.macieja@umed.lodz.pl (A.M.); piotr.machnicki@umed.lodz.pl (P.M.); dorota.pastuszak-lewandoska@umed.lodz.pl (D.P.-L.); 2 Medical Microbiology Laboratory, Central Teaching Hospital of Medical University of Lodz, 92-213 Lodz, Poland

**Keywords:** antimicrobial susceptibility, extended-spectrum β-lactamase, genetic epidemiology, Enterobacterales, antimicrobial resistance genes

## Abstract

The extended-spectrum β-lactamases (ESβLs) are bacterial enzymes capable of hydrolyzing penicillins, cephalosporins, and aztreonam. The prevalence of ESβL is increasing among clinically significant microorganisms worldwide, drastically reducing the therapeutic management of infectious diseases. The study aimed to determine the drug susceptibility of ESβL-positive clinical isolates acquired from patients hospitalized in Lodz, central Poland, and analyze the prevalence of specific genes, determining acquired resistance in these bacteria. The samples of ESβL-positive clinical isolates were gathered in 2022 from medical microbiological laboratories in the city of Lodz, central Poland. The strains were subjected to biochemical identification and antimicrobial susceptibility testing following EUCAST guidelines. The presence of studied genes (bla*_CTX-M_*, bla*_SHV_*, bla*_TEM_*, bla*_PER_*, bla*_VEB_*) was confirmed by PCR. Over 50% of studied isolates were resistant to gentamicin, cefepime, ceftazidime and ciprofloxacin. The most common ESβL gene was bla*_CTX-M_*. In most isolates, the resistance genes occurred simultaneously. The bla*_PER_* was not detected in any of the tested strains. ESβL-producing strains are largely susceptible to the currently available antibiotics. The observation of the coexistence of different genes in most clinical isolates is alarming.

## 1. Introduction

The extended-spectrum β-lactamases (ESβLs) are β-lactamases containing a serine in the active site, which belong to class A or D according to the Ambler classification and group 2 according to the Bush–Jacoby classification [1,2]. They are capable of hydrolyzing penicillins, cephalosporins (including third and fourth generation forms), and aztreonam. They do not hydrolyze carbapenems, temocillin or cephamycins, such as cefoxitin, and are inactivated in vitro by β-lactamase inhibitors (clavulanic acid, tazobactam, and sulbactam, vaborbactam, relebactam, avibactam). Their structural genes are found in mobile genetic elements, such as plasmids, integrons, and transposons, which are capable of transfer within and between species [3].

β-lactamase production is a natural process. Most ESβLs result from a single mutation in the active site of typical β-lactamases (TEM-1, TEM-2, and SHV-1), while newer forms (CTX-M) are derived from cephalosporinases of certain plant bacterial strains (*Kluyvera* spp.), which are then incorporated into mobile genetic elements. There are currently over 350 different ESβLs, and numerous unrelated enzymes have been described (e.g., OXA, CTX-M, PER, VEB, GES, BES, TLA, SFO, and IBC) [4].

The *Klebsiella pneumoniae* strain producing ESβL was first isolated in Germany in 1983. Later, the introduction of oxyimino-β-lactams, i.e., cefuroxime and third-generation cephalosporins, resulted in the emergence of ESβL-type β-lactamases [4,5]. Such ESβLs are currently a source of serious clinical and epidemiological problems worldwide, and although they are most commonly observed in nosocomial strains of Enterobacterales bacilli, their prevalence has been increasing in strains causing community-acquired infections, particularly *Escherichia coli* [6].

The prevalence of ESβL is also increasing among clinically significant microorganisms; studies performed in 13 regional secondary-care hospitals in Poland have found them to be present in 40–60% of *K. pneumoniae* clinical isolates in a single department or hospital, which consistently narrows down the therapeutic possibilities of β-lactam antibiotics [7]. Although various methods can be used to detect ESβL, all are generally based on recording a significant decrease in the susceptibility to at least one marker oxyimino-β-lactam (ceftazidime, cefotaxime, ceftriaxone, cefpodoxime, cefepime, aztreonam) and the demonstration of the effect of the β-lactamase inhibitor (clavulanic acid) for this effect [8].

Unfortunately, the diagnosis of ESβL-positive strains is hampered by the fact that they demonstrate a wide range of resistance phenotypes to β-lactams, being in vitro susceptible to selected ESβL substrates. More worryingly, the genes encoding ESβL are located on plasmids which usually also contain genes that determine resistance to inter alia aminoglycosides [9]; hence, they are often resistant to antibiotics from other therapeutic groups, making it even easier for them to undergo positive selection, persist in the hospital flora and cause clonal epidemics.

The aim of the study was to determine the drug susceptibility of ESβL-positive Gram-negative bacilli isolated from clinical samples acquired from patients hospitalized in Lodz, central Poland, and analyze the prevalence of specific genes (bla*_CTX-M_*, bla*_SHV_*, bla*_TEM_*, bla*_PER_*, bla*_VEB_*), determining acquired resistance in these strains.

## 2. Results

### 2.1. Antimicrobial Susceptibility

The studied ESβL-producing Gram-negative bacteria were found to be 53.1% susceptible to piperacillin/tazobactam, 4.1% to cefepime, and 9.2% to the third-generation cephalosporin ceftazidime. A high percentage of isolates were susceptible to cefiderocol (99%) and ceftazidime/avibactam (95%), as well as to the carbapenems imipenem (93%) and meropenem (97%). They were also found to be highly susceptible to combinations of carbapenems with new non-β-lactam inhibitors, such as meropenem/vaborbactam and imipenem/relebactam (95% and 96%, respectively). Only 5.1% of ESβL-positive strains were susceptible to ciprofloxacin, which was in line with the strong level of resistance demonstrated to the fluoroquinolone group. Among the aminoglycosides, 89% of isolates were susceptible to amikacin and only 39% to gentamicin; in addition, 92% of strains were susceptible to colistin.

Among *E. coli* isolates producing ESβLs, 91% were susceptible to piperacillin with tazobactam, 7% were susceptible to cefepime, and only 11.4% to ceftazidime. A high percentage of isolates were susceptible to cefiderocol and ceftazidime/avibactam (98%). All of the isolates were susceptible to carbapenems, such as imipenem and meropenem. They were also highly susceptible to combinations of carbapenems with novel inhibitors, such as meropenem/vaborbactam and imipenem/relebactam (98%). Only 4.5% of strains were susceptible to ciprofloxacin. In contrast, 91% were susceptible to amikacin, 52.3% to gentamicin, 98% to colistin and 89% to nitrofurantoin.

The tested *K. pneumoniae* species showed only 23.1% susceptibility to piperacillin with tazobactam, 88.5% resistance to cefepime, and 100% resistance to ceftazidime. All isolates were susceptible to cefiderocol, 88.5% to ceftazidime/avibactam, and 92.3% to the carbapenems imipenem and meropenem. In addition, 88.5% of the *K. pneumoniae* isolates were susceptible to meropenem/vaborbactam, and 92.3% to imipenem/relebactam. Only 8% were susceptible to ciprofloxacin. Regarding the aminoglycosides, 88.5% of isolates were susceptible to amikacin and 50% to gentamicin. Finally, 96.2% of ESβL-positive *K. pneumoniae* strains were susceptible to colistin.

Among the remaining Gram-negative bacteria (CESP group—*Citrobacter* spp., *Enterobacter* spp., *Serratia* spp., *Proteus* spp.), only 21.4% were susceptible to piperacillin/tazobactam, 14.3% to ceftazidime and none to cefepime. All isolates were susceptible to cefiderocol, 96.4% to ceftazidime/avibactam, 82.1% to imipenem and 96.4% to meropenem, the latter two being carbapenem antibiotics. In addition, 96.4% of strains were susceptible to combinations of carbapenems with novel non-β-lactam inhibitors. Fewer than 4% of strains were susceptible to ciprofloxacin. Otherwise, 86% of isolates were susceptible to amikacin and only 7.1% to gentamicin, while 78.6% of ESβL-positive strains were susceptible to colistin, excluding naturally resistant species.

**Figure 1:**
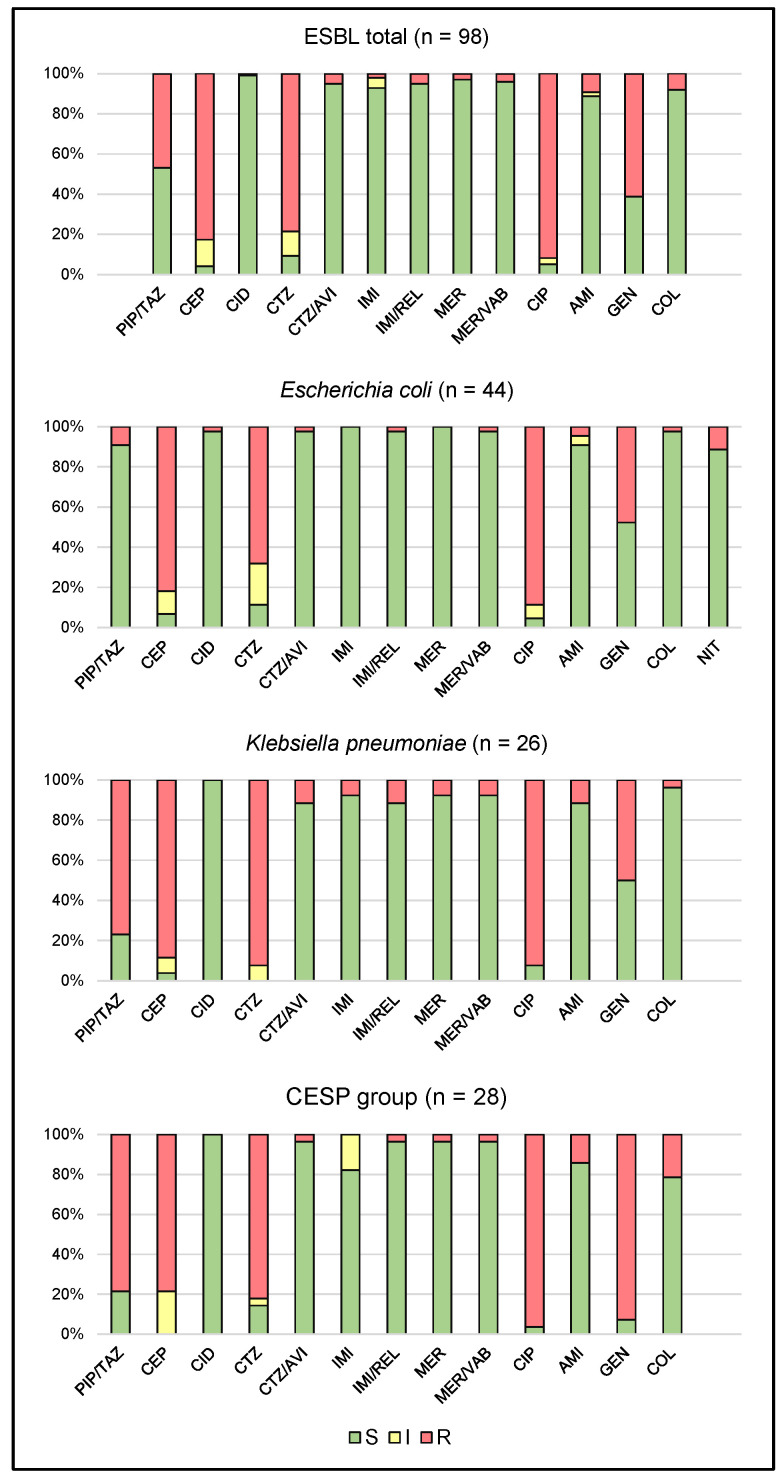
Drug susceptibility of the studied strains producing extended-spectrum beta-lactamases (CESP group—*Citrobacter* spp., *Enterobacter* spp., *Serratia* spp., *Proteus* spp.; S—susceptible; I—susceptible, increased exposure; R—resistant; PIP/TAZ—piperacillin/tazobactam; CEP—cefepime; CID—cefiderocol; CTZ—ceftazidime; CTZ/AVI—ceftazidime/avibactam; IMI—imipenem; IMI/REL—imipenem/relebactam; MER—meropenem; MER/VAB—meropenem/vaborbactam; CIP—ciprofloxacin; AMI—amikacin; GEN—gentamicin; COL—colistin; NIT—nitrofurantoin).

The drug susceptibility of the studied ESβL clinical isolates is presented in Figure 1.

### 2.2. Genetic Epidemiology

The most commonly identified gene was bla*_CTX-M_*; being unaccompanied in 9.2% of cases and accompanied with bla*_SHV_* in 3.1% of isolates and with bla*_SHV_* and bla*_TEM_* in 21.5% simultaneously. The bla*_CTX-M_*, bla*_SHV_*, bla*_TEM_*, and bla*_VEB_* genes were detected in 2% of isolates; bla*_TEM_* was unaccompanied in 5.1% of cases, with bla*_VEB_* in 1%, and with bla*_CTX-M_* and bla*_VEB_* in 8.2%. The bla*_SHV_* was present alone in 4.1% of strains, and together with bla*_TEM_* or bla*_VEB_* in 1% of bacteria. The bla*_TEM_* was detected alone in 5.1% of isolates, and bla*_TEM_* and bla*_VEB_* in 1%. bla*_VEB_* alone was confirmed in 2% of cases. Among the tested strains, 26.5% demonstrated none of the tested ESβL genes.

Regarding the isolated species, among *E. coli* strains, the most common ESβL gene was bla*_CTX-M_* (alone or with other genes), which was observed in 27.3% of cases. The second most abundant type was bla*_TEM_* (11.4%), followed by bla*_SHV_* (6.8%) and bla*_VEB_* (2.3%)—each of them alone. In 52.2% of the strains, none of the tested genes were observed.

Among *K. pneumoniae*, as in the case of *E. coli*, the most common gene was bla*_CTX-M_* ESβL (alone or with other genes), observed in 88.6% of cases, followed by bla*_SHV_* (11.4%), bla*_SHV_* gene alone (3.8%), bla*_SHV_* with bla*_TEM_* (3.8%), and bla*_SHV_* with bla*_VEB_* (3.8%).

Among the remaining isolated Gram-negative bacteria (CESP group), bla*_CTX-M_* was most often observed, being detected alone or with other genes in 82.1% of cases. This was followed by bla*_VEB_* alone (3.6%) and bla*_VEB_* with bla*_TEM_* (3.6%). In 10.6% of the strains, none of the tested genes were found.

**Figure 2:**
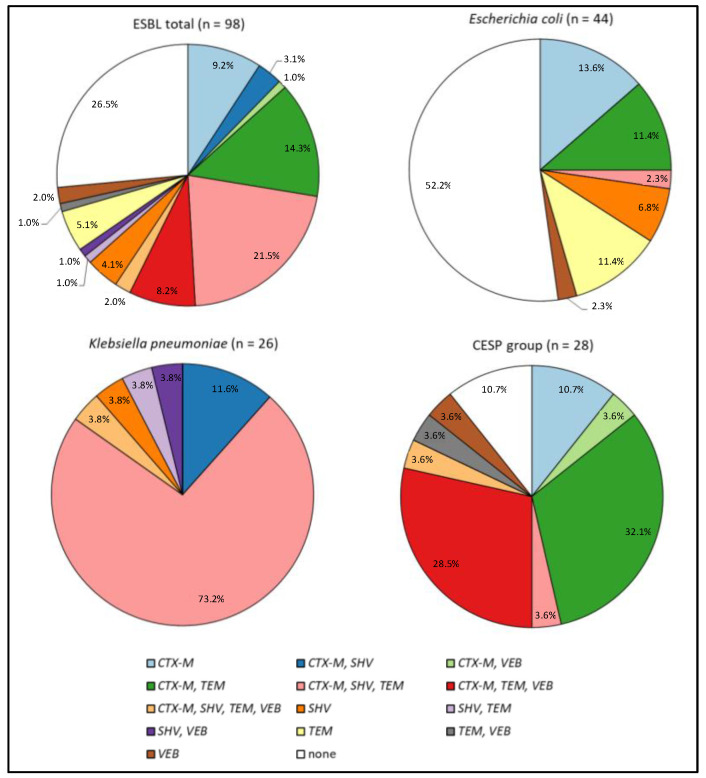
The percentage of the occurrence of various ESβL genes (bla*_CTX-M_*, bla*_SHV_*, bla*_TEM_*, bla*_PER_*, bla*_VEB_*) in the studied clinical isolates.

The percentage of the occurrence of ESβL genes in the studied clinical isolates is presented in Figure 2.

## 3. Discussion

### 3.1. Antimicrobial Susceptibility

During the period under study, ESβL production was most frequently observed among *K. pneumoniae* and *E. coli* strains, which constituted the majority of the examined set of bacterial isolates. Similar results have been obtained in previous studies [10], which, like the present study, also note that the strains display high resistance to ceftazidime and gentamicin, and high susceptibility to carbapenems (over 90%). However, considerably greater fluoroquinolone resistance was noted in the present study (34% vs. 5.1%).

Growing resistance to fluoroquinolones has been observed, posing a significant public health challenge [11]. Unfortunately, the overuse of these drugs in treating infections has reduced their effectiveness against Gram-negative Enterobacterales. Our findings indicate that ESβL-positive strains demonstrate strong resistance to ciprofloxacin: over 95% of *E. coli* and 92% of *K. pneumoniae*. Similarly, Talan et al. [12] showed strong resistance to ciprofloxacin (81%) and similarly to the present studies, high resistance to gentamicin (over 40%).

The rapid growth of drug resistance and the spread of bacteria able to transmit acquired resistance in the hospital environment have negated the use of a number of drugs previously used in therapy. New combinations with inhibitors such as meropenem/vaborbactam, imipenem/relebactam, and ceftazidime/avibactam may be an alternative in the treatment of severe infections, e.g., those caused by ESβL-positive and carbapenem-resistant strains. These antimicrobials have been found to be very effective against β-lactam-resistant strains [13]; similarly, in our study the isolates demonstrated over 90% susceptibility to these new antibiotics. Our findings also showed that cefiderocol, a new siderophore cephalosporin, may be a potential alternative in treating infections caused by multidrug-resistant strains. Similar conclusions were reached by Kaye et al. [14]. However, it should be clearly emphasized that as we analyzed only previously selected ESβL-positive isolates, this resulted in increased rates of antimicrobial resistance.

There is a need for further assessment of the susceptibility of ESβL-positive strains to new antibiotics intended to treat infections caused by multidrug-resistant strains in routine microbiological diagnostics. It should also be borne in mind that carbapenemase-producing organisms (CPO) almost always produce one or more other β-lactamases, often including ESβL. The combined activity of all these enzymes results in β-lactam resistance. Relatively few organisms producing metallo-β-lactamase are susceptible to aztreonam due to the common presence of ESβL or AmpC β-lactamases [15,16]. For this reason, the choice of effective treatment is difficult.

ESβL is a less critical problem in terms of treatment alternatives than infections caused by CPO. There is a need to assess the effectiveness of both the basic antibiotics used to treat these infections as well as antibiotics reserved for CPO. Our findings indicate that many drugs used successfully in Gram-negative infections may be ineffective against ESβL-positive bacteria, and often only new antimicrobial drugs can be used. This is echoed by Bassetti et al. [17].

### 3.2. Genetic Epidemiology

The dominant ESβL gene in our region (central Poland) was found to be bla*_CTX-M_*.

Bastidas-Caldes et al. found the most common β-lactamase and mobilized colistin resistance (MCR) enzyme genes associated with ESβL-based acquired resistance in *E. coli* to be bla*_TEM_*, followed by bla*_CTX-M_*. In *K. pneumoniae*, bla*_SHV_* and bla*_TEM_* predominated, followed by bla*_CTX-M_* [18].

Also, Masoud et al. [19] found bla*_TEM_* to be the most common resistance gene among the ESβL-positive strains, followed by bla*_SHV_* and bla*_CTX-M_*. In addition, most isolates contained more than one resistance gene (81%).

In *K. pneumoniae*, Kazemian et al. [20] reported that eight isolates carried two, and three isolates had three ESβL genes. They also noted the simultaneous presence of two ESβL genes in six *E. coli* strains, and three genes in three of them. In addition, none of their strains were found to carry the bla*_PER_* gene, and the bla*_CTX-M_* was most common.

Similar conclusions were reached by Irfan et al. [21], who reported multiple occurrences of ESβL genes in *E. coli* and *K. pneumoniae* and the dominance of the bla*_CTX-M_* in the tested isolates.

Deku et al. [22] reported bla*_TEM_* gene to be the dominant ESβL genotype in confirmed *E. coli* isolates (83.9%), with bla*_CTX-M_* and bla*_SHV_* detected in 69.6% and 35.7%, respectively. In our present study, 16 ESβL-producing isolates were found to have a single ESβL-encoding gene, while most isolates had two different ESβL genes, with three isolates carrying four different ESβL genes. Fifteen of the fifty-six *E. coli* isolates contained the bla*_TEM_* and bla*_CTX-M_* genes.

Our findings appear to be in line with most of the literature. Previous research indicates that both ESβL-type resistance genes and those conferring resistance to carbapenems and colistin in Enterobacterales pose a significant threat to public health. Of particular concern is the presence of these kinds of strains in animals bred for human consumption, which may contribute to the transmission of multidrug-resistant strains to humans [23].

## 4. Materials and Methods

A total of 98 Gram-negative bacilli clinical strains producing ESβL were investigated—44 *E. coli*, 26 *K. pneumoniae*, and 28 CESP group bacteria. All were taken from the collection of the Department of Microbiology and Laboratory Medical Immunology; the samples were gathered in the year 2022 from medical microbiological laboratories in the city of Lodz, central Poland. All strains were isolated from the following clinical samples: bronchial alveolar lavage (BAL, n = 4), blood (n = 13), urine (n = 43), rectal swab (resistance mechanism carriage screening, n = 17), lower respiratory specimen (other than BAL, n = 2), intraoperative swab (n = 5), nasal swab (n = 2), wound swab (n = 6), and pressure ulcer swab (n = 6).

All bacteria were stored in Viabank storage beads (Medical Wire and Equipment, Corsham, UK) at a maximum of −80 °C for six months and regenerated on Columbia Agar with 5% sheep blood (Thermo Fisher Scientific, Waltham, MA, USA) for 18–24 h at 37 °C. The strains were subjected to biochemical identification and a drug susceptibility assessment using an automated BD Phoenix system (Becton Dickinson and Company, Franklin Lakes, NJ, USA). Colistin susceptibility was assessed using the MICRONAUT MIC-Strip colistin assay (MERLIN Diagnostika, Bornheim, Germany). Cefiderocol susceptibility was assessed using the disc diffusion method with 30 μg antibiotic discs (Oxoid, Wesel, Germany). The isolates were also tested for susceptibility to meropenem/vaborbactam, imipenem/relebactam, and ceftazidime/avibactam using minimum inhibitory concentration (MIC) test gradient strips (Liofilchem, Roseto degli Abruzzi, Italy). Drug susceptibility was determined on standard Mueller–Hinton Agar (Thermo Fisher Scientific, Waltham, MA, USA), incubated for 18 ± 2 h at 35 ± 1 °C following the European Committee on Antimicrobial Susceptibility Testing (EUCAST) guidelines [24].

ESβL production was determined by the double-disc synergy test (DDST) using cefotaxime (30 µg), ceftazidime (30 µg) and amoxicillin/clavulanic acid (30 µg). The amoxicillin/clavulanic acid disc was placed no further than 2 cm from the cefotaxime and ceftazidime discs, and the culture was incubated for 18 ± 2 h at 35 ± 1 °C. ESβL production was proved by a markedly enlarged zone of inhibition around the cefotaxime or ceftazidime disc on the side of the amoxicillin/clavulanic acid disc [6].

The presence of the studied genes (bla*_CTX-M_*, bla*_SHV_*, bla*_TEM_*, bla*_PER_*, bla*_VEB_*) was confirmed by PCR. Bacterial genomic DNA was obtained using the Genomic Mini AX Bacteria Spin kit (A&A Biotechnology, Gdansk, Poland), according to the manufacturer’s instructions. DNA was amplified using HotStarTaq Master Mix Kit (Qiagen, Venlo, The Netherlands): the primer sequences for each gene are listed in Table 1. PCRs were performed as follows: initial denaturation 95 °C for 15 min, followed by 35 cycles of denaturation (94 °C for 30 s), annealing (50 °C for 30 s), and extension (72 °C for 30 s). After the last step, a final extension was performed at 72 °C for three minutes. The same conditions were used for all the genes. The PCR products were separated in a 2% agarose gel for 60 min with a voltage of 7 V/cm. All reactions were performed in the presence of a negative control, K(-)MIX, which gave negative results. GeneRuler 100 bp Plus (Thermo Fisher Scientific, Waltham, MA, USA) was used as a DNA size marker. Gel visualization after electrophoresis was performed on BioDoc-It™ UVP^®^ (Analytik Jena, Jena, Germany). The example of PCR photo is presented in Supplementary Materials, Figure S1.

Descriptive statistics were prepared using Microsoft Excel 2019 software (Microsoft Corporation, Redmond, WA, USA).

### 4.1. Ethical Issues

The presented research was conducted with high ethical standards. All patient data were anonymized to prevent any possibility of identifying a specific participant. All bacterial strains had been previously secured in the culture collection of the research unit and encoded using consecutive identification numbers. The only available clinical data concerned the sex and age of the patients and the type of biological material from which the bacterial strain was isolated.

### 4.2. Limitations

One of the limitations of the study was the relatively low number of tested isolates—they were not collected from all hospitals in the region, but only from the 3 largest medical centres. However, the obtained data undoubtedly paint a picture of the actual situation in the discussed topic.

The other limitation was the lack of sequential analysis and detection of specific mutations conferring the ESβL phenotype. Thus, when writing about ESβL strains, we mean their phenotypically confirmed status; while gene identification draws attention to the type of resistance, answering the question of which of them predominates.

The detection of ESBL strains using phenotypic methods in accordance with the recommendations, both CLSI and EUCAST, is a screening test which has many limitations, e.g., false negative results may occur due to the co-production of AmpC β-lactamase. Nevertheless, it is a method that leads to the reduction in ineffective therapy in the case of infections caused by these strains. Molecular techniques, such as DNA microarray assays, PCR and/or sequencing and WGS—which are used in some research studies—are not implemented in clinical laboratories due to the complexity and high costs of the methods, as well as the requirement of highly trained staff with molecular and/or bioinformatics expertise.

Finally, strains isolated from rectal swabs were also included, but it is important to point out that they are defined as colonization, and antimicrobial treatment should not be used in this situation.

## 5. Conclusions

In conclusion, the study highlights the problem of the significant prevalence and complexity of antimicrobial resistance among ESβL-producing Enterobacterales in central Poland. The high resistance rates to commonly used antimicrobials, including gentamicin, ceftazidime, and ciprofloxacin, underline the critical need for ongoing surveillance and novel treatment strategies. The effectiveness of newer antibiotics, such as cefiderocol and combinations like meropenem/vaborbactam, provides some hope, yet the co-occurrence of multiple resistance genes in most isolates poses a continuous threat to public health. The identification of genes determining a specific type of resistance allowed us to find out which of them predominates in hospitals in the studied geographical region. This type of knowledge has a specific impact on the use of antimicrobials in empirical therapy, but also—due to its incompleteness—it emphasizes the need for the further research, continuous epidemiological control and surveillance of the genes encoding ESβL-type resistance.

## Figures and Tables

**Table 1:** Primer sequences for each gene considered in the study.

Gene or Region	Primers (5′ → 3′)	Amplicon Size [bp]	Reference
bla*_CTX-M_*	F: ATGTGCACCAGTAARGT R: TGGGTRAARTARGTSACCAGA	593	[25]
bla*_SHV_*	F: ATTTGTCGCTTCTTTACTCGCC R: TTCACCACCATCATTACCGACC	1027	[26]
bla*_TEM_*	F: GTGCGCGGAACCCCTATT R: GGGATTTTGGTCATGAGATTATC	1083	[26]
bla*_PER_*	F: AATTTGGGCTTAGGGCAGAA R: ATGAATGTCATTATAAAAGC	924	[26]
bla*_VEB_*	F: CGACTTCCATTTCCCGATGC R: GGACTCTGCAACAAATACGC	642	[27]

## Data Availability

The data presented in this study are available on request from the corresponding author.

## Supplementary Materials

The following supporting information can be downloaded at: https://www.mdpi.com/article/10.3390/ijms25158371/s1.

## References

[1] Bush K., Jacoby G.A. (2010). Updated functional classification of beta-lactamases. Antimicrob. Agents Chemother..

[2] Ambler R.P., Coulson A.F., Frère J.M., Ghuysen J.M., Joris B., Forsman M., Levesque R.C., Tiraby G., Waley S.G. (1991). A standard numbering scheme for the class A beta-lactamases. Biochem. J..

[3] Zurfluh K., Hächler H., Nüesch-Inderbinen M., Stephan R. (2013). Characteristics of extended-spectrum β-lactamase- and carbapenemase-producing *Enterobacteriaceae* isolates from rivers and lakes in Switzerland. Appl. Environ. Microbiol..

[4] Castanheira M., Simner P.J., Bradford P.A. (2021). Extended-spectrum β-lactamases: An update on their characteristics, epidemiology and detection. JAC Antimicrob. Resist..

[5] Knothe H., Shah P., Krcmery V., Antal M., Mitsuhashi S. (1983). Transferable resistance to cefotaxime, cefoxitin, cefamandole and cefuroxime in clinical isolates of *Klebsiella pneumoniae* and *Serratia marcescens*. Infection.

[6] Cantón R., Novais A., Valverde A., Machado E., Peixe L., Baquero F., Coque T.M. (2008). Prevalence and spread of extended-spectrum beta-lactamase-producing *Enterobacteriaceae* in Europe. Clin. Microbiol. Infect..

[7] Empel J., Baraniak A., Literacka E., Mrówka A., Fiett J., Sadowy E., Hryniewicz W., Gniadkowski M. (2008). Molecular survey of beta-lactamases conferring resistance to newer beta-lactams in *Enterobacteriaceae* isolates from Polish hospitals. Antimicrob. Agents Chemother..

[8] Drieux L., Brossier F., Sougakoff W., Jarlier V. (2008). Phenotypic detection of extended-spectrum beta-lactamase production in *Enterobacteriaceae*: Review and bench guide. Clin. Microbiol. Infect..

[9] Bush K., Bradford P.A. (2016). β-Lactams and β-lactamase inhibitors: An overview. Cold Spring Harb. Perspect. Med..

[10] Onduru O.G., Aboud S., Nyirenda T.S., Rumisha S.F., Mkakosya R.S. (2021). Antimicrobial susceptibility testing profiles of ESBL-producing Enterobacterales isolated from hospital and community adult patients in Blantyre, Malawi. IJID Reg..

[11] WHO/ECDC, Antimicrobial Resistance Surveillance in Europe 2022–2020 Data. https://www.ecdc.europa.eu/sites/default/files/documents/Joint-WHO-ECDC-AMR-report-2022.pdf.

[12] Talan D.A., Takhar S.S., Krishnadasan A., Abrahamian F.M., Mower W.R., Moran G.J. (2016). EMERGEncy ID Net Study Group. Fluoroquinolone-resistant and extended-spectrum β-lactamase-producing *Escherichia coli* infections in patients with pyelonephritis, United States. Emerg. Infect. Dis..

[13] Yahav D., Giske C.G., Grāmatniece A., Abodakpi H., Tam V.H., Leibovici L. (2020). New β-lactam-β-lactamase inhibitor combinations. Clin. Microbiol. Rev..

[14] Kaye K.S., Naas T., Pogue J.M., Rossolini G.M. (2023). Cefiderocol, a siderophore cephalosporin as a treatment option for infections caused by carbapenem-resistant Enterobacterales. Infect. Dis. Ther..

[15] Boyd S.E., Livermore D.M., Hooper D.C., Hope W.W. (2020). Metallo-β-lactamases: Structure, function, epidemiology, treatment options and the development pipeline. Antimicrob. Agents Chemother..

[16] Bush K., Bradford P.A. (2020). Epidemiology of β-lactamase-producing pathogens. Clin. Microbiol. Rev..

[17] Bassetti M., Righi E. (2015). New antibiotics and antimicrobial combination therapy for the treatment of gram-negative bacterial infections. Curr. Opin. Crit. Care.

[18] Bastidas-Caldes C., Cisneros-Vásquez E., Zambrano A., Mosquera-Maza A., Calero-Cáceres W., Rey J., Yamamoto Y., Yamamoto M., Calvopina M., de Waard J.H. (2023). Co-harboring of beta-lactamases and mcr-1 genes in *Escherichia coli* and *Klebsiella pneumoniae* from healthy carriers and backyard animals in rural communities in Ecuador. Antibiotics.

[19] Masoud S.M., Abd El-Baky R.M., Aly S.A., Ibrahem R.A. (2021). Co-existence of certain ESBLs, MBLs and plasmid mediated quinolone resistance genes among MDR *E. coli* isolated from different clinical specimens in Egypt. Antibiotics.

[20] Kazemian H., Heidari H., Ghanavati R., Ghafourian S., Yazdani F., Sadeghifard N., Pakzad I. (2019). Phenotypic and genotypic characterization of ESBL-, AmpC-, and carbapenemase-producing *Klebsiella pneumoniae* and *Escherichia coli* isolates. Med. Princ. Pract..

[21] Irfan S., Azhar A., Bashir A., Ahmed S., Haque A. (2021). High frequency of simultaneous presence of ESBL and carbapenemase producers among nosocomial coliform isolates in Faisalabad, Pakistan. Pak. J. Med. Sci..

[22] Deku J.G., Duedu K.O., Ativi E., Kpene G.E., Feglo P.K. (2021). Occurrence and distribution of extended-spectrum β-lactamase in clinical *Escherichia coli* isolates at Ho Teaching Hospital in Ghana. Ghana. Med. J..

[23] Bastidas-Caldes C., Guerrero-Freire S., Ortuño-Gutiérrez N., Sunyoto T., Gomes-Dias C.A., Ramírez M.S., Calero-Caceres W., Harries A.D., Rey J., de Waard J.H. (2023). Colistin resistance in *Escherichia coli* and *Klebsiella pneumoniae* in humans and backyard animals in Ecuador. Rev. Panam. Salud Publica.

[24] EUCAST Breakpoint Tables for Interpretation of MICs and Zone Diameters, Ver. 14.0. https://www.eucast.org/fileadmin/src/media/PDFs/EUCAST_files/Breakpoint_tables/v_14.0_Breakpoint_Tables.pdf.

[25] Moghaddam M.N., Beidokhti M.H., Jamehdar S.A., Ghahraman M. (2014). Genetic properties of bla*CTX-M.* and bla*PER* β-lactamase genes in clinical isolates of *Enterobacteriaceae* by polymerase chain reaction. Iran. J. Basic. Med. Sci..

[26] Ejaz H. (2020). Dissemination of *SHV*, *TEM* and *CTX-M.* genotypes in *Pseudomonas aeruginosa*: A pre-eminent reason for therapeutic failure in pediatrics. Ann. Clin. Lab. Sci..

[27] Amirkamali S., Naserpour-Farivar T., Azarhoosh K., Peymani A. (2017). Distribution of the bla*OXA*, bla*VEB-1*, and bla*GES-1* genes and resistance patterns of ESBL-producing *Pseudomonas aeruginosa* isolated from hospitals in Tehran and Qazvin, Iran. Rev. Soc. Bras. Med. Trop..

