# Supplementary figures and images for: Antimicrobial Susceptibility and Genetic Epidemiology of Extended-Spectrum β-Lactamase-Positive Enterobacterales Clinical Isolates in Central Poland
Source: Int J Mol Sci. 2024 Jul 31;25(15):8371. doi: 10.3390/ijms25158371 (PMC11312491; doi:10.3390/ijms25158371)

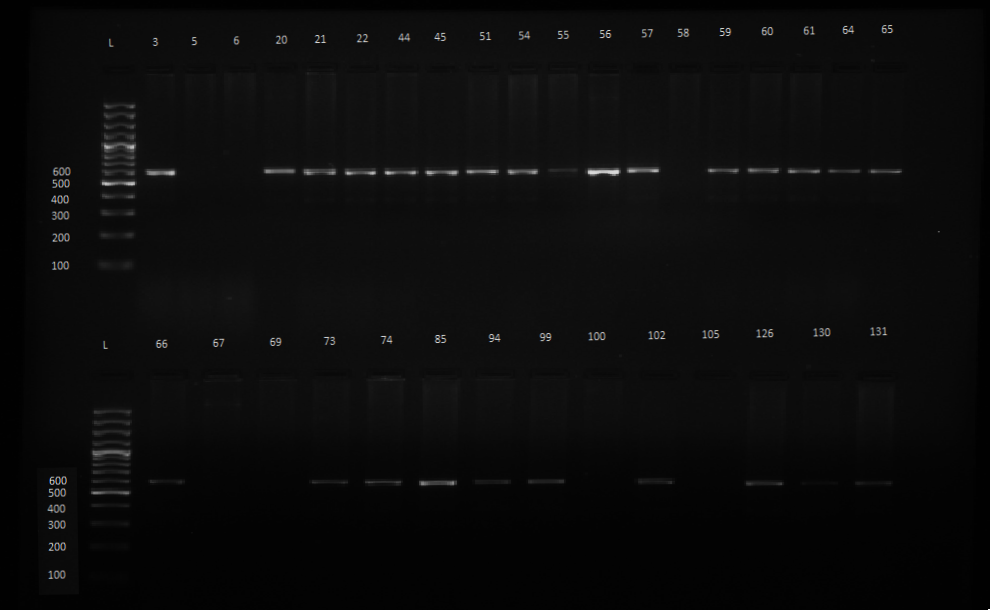

Supplement: Supplementary file 1 [file ijms-25-08371-s001.zip › ijms-3105771-supplementary.png]
